# Round the Hospitals

**Published:** 1917-02-03

**Authors:** 


### ROUND THE HOSPITALS.

In The Hospital of January 20, page 327, we dealt with Homeland Workers in Wartime, and insisted that the remedy of recognition was an imperative duty. There is one fact which is not understood by the general public, nor fully realised by those responsible for the distribution of honours, which requires enforcement at the present time. It is that war brings a restlessness of trained workers, to whom it acts as a bugle-call more than to others, and, in the case of women workers, makes every one of them wish to be at the Front. Now no one who has had much to do with the organisation for the present war will deny that the secret of success in wartime, in whatever part of the world, rests mainly on the efficiency of the organisation at home —that is, at the centre—where those who work, arrange, organise, and maintain supplies of every kind, have, in the case of women, to overcome the wish just mentioned and settle down to continuous, laborious, and largely unrecognised work of the most valuable kind by toiling in an office under a matron-in-chief. We are glad to hear that since our article was written a move is probable in the direction of recognition for this important type of woman worker. There can be no doubt that it is generally recognised that in the case of the bestowal of the Royal Red Cross the fault so far has been to give it too freely, and to confine it mainly to those who have had a measure, at any rate, of foreign service. It is true that some home workers in the Territorial system have been rewarded by the bestowal of the Royal Red Cross, but there yet remain a few, and amongst them some of the ablest matrons, who have laboured assiduously under the matrons-in-chief at one or more of the central offices, who have not yet received any decoration. In their case we ventured to point out that they, of all women workers, are fully entitled to the honour of being decorated with the Royal Red Cross.

It has been laid down, we understand, as a general principle that home workers, especially in the case of men, are not to have any decoration or other recognition until the termination of the war. This regulation, made we believe at the outset of the war, experience proves to offer drawbacks which, in view of the invaluable character of much of the home service rendered by a multitude of people, points to injustice, and even to a risk of a lower efficiency than might otherwise be the case. So it has come to be recognised that a new decoration may be instituted which will enable service of the kind indicated to be acknowledged before the termination of the war. If so, a new honour may be open to all workers throughout the Empire. An Order of the British Empire, if instituted, would seem to be an appropriate means of conferring honour and recognition on the multitude of workers referred to, amongst whom the V.A.D.s will necessarily have an honourable claim. Such an Order would not, however, be suitable to meet the case of the able matrons working under a matron-in-chief, whom we have in view. For them, if justice

**ROUND THE HOSPITALS**— (*continued*).

is to be done, the grant of the Royal Red Cross is the one just recognition, having regard to the distribution that has already been made of this Order to matrons, sisters, and nurses, and to the special services and great self-denial and patriotism exhibited by those matrons, relatively few in number, who have given such invaluable service to the nation throughout the war, at the central offices at home, as assistants to the matrons-in-chief.

At the great meeting at Leeds on the 19th ultimo (see The Hospital for January 27, page 348), Miss Swift made some statements which answered clearly several questions which nurses are putting at the present time. Miss Swift properly declared it to be very important to the nursing profession that there should be uniformity of qualification secured by uniformity of curriculum and a single-portal examination. At present qualified nurses had no legal status and that was why, they wanted every nurse to join the Royal British College of Nursing, to make effective the demand for the State Registration of trained nurses. At the expiration of the period of grace, two years, nurses who had had a two years' training only would not be recognised as trained nurses. Miss Swift declared that training in a military hospital did not count. There were a great many helpers in this war, V.A.D. and others, without whom the work of the war could not have been carried on, but their experience could not count to them in the future. That made their present sacrifice greater. A military hospital was a special hospital. Miss Swift finally stated that there would be a Supplementary Register of Nurses who had devoted themselves to work Amongst Children. All these matters are important. We hope the College will speedily prepare and issue a complete and up-to-date statement covering the requirements and present position of the College, towards Trained Nurses, and of Nurses Generally, towards the College.

The nursing profession and all well-wishers of the Royal British College of Nursing owe grateful thanks to Miss Cox-Davies and Miss Rundle for their energy and courage in facing the many incidental discomforts of travel in the trying weather of the last few weeks, in order to attend large meetings of practitioners and nurses in Ireland (see page 372). Their public spirit appears to have been gratefully recognised by the large attendances at the meetings held in Dublin and Belfast at which they were present, and their efforts to explain the true aims of the Royal British College of Nursing, and the advantages which it offers to all fully-trained nurses and to the nursing profession as a whole, seem to have been largely successful. In one sense the meetings might have been held in Scotland, where heckling has become a fine art, if we may judge by the continuous string of questions from all parts of the hall, the nature of these questions, and the opposing views and wishes of the questioners, which they exhibit. It is not possible to give these questions and answers in any complete sense this week. We have therefore thought it best to hold them over, that the mind of the Irish speakers and questioners may be fully demonstrated by the publication, of as complete a report as possible, of the whole of these questions and answers.

We would specially draw the attention of our readers this week to the bogus charge of the party of "Desire to kill" or "Divide" that the professional status of the Royal British Nurses' Association will, under the Supplemental Charter, be absorbed into a lay corporation. In the article published on page 369, to which we invite our readers' attention, the charge of lay control is proved to be a myth. It will be seen that it is not the Royal British College of Nursing that is in danger of falling under lay control, but in reality those who put their faith in the Central Committee for the State Registration of Nurses, as a close perusal of the latest edition of the latter's Registration Bill demonstrates.

There is a fund of good material which, if garnered and published in The Hospital, could not fail to excite wide interest and do good to workers and hospital supporters throughout the nation. We have in the past published some very good, crisp stories of incidents of the kind, and the war has multiplied the incidents and the stories indefinitely. Each should be compressed into a short paragraph not exceeding twenty lines or 150 words or so. The sister, who is ever on guard and always alert, is the centre to which we look mainly for copy of this kind. We are prepared to give 5s. for each such story which we may be able to accept and publish in The Hospital. We hope some of our readers at any rate will turn their attention to this matter and that the result will prove helpful, instructive, and amusing to all workers and readers. Amongst the V.A.D.s are a very large number of able and highly educated women, and possibly they, or some of them, coming fresh to the work, might create a new kind of story, which would add to the interest and give stimulus to the effort we have ventured to invite. The following story was sent us recently, and we give it by way of illustration:

Miles (age three): "Sister!" Sister: "Yes, sonny." Miles: "Look what I've got," producing a bundle of small pictures of celebrities collected from cigarette packets. After sister had duly admired his treasures, Miles held one of King Edward before her and said, "I am going to keep this to show the Queen." Sister smiled, and the incident passed from her mind. A fortnight later, on the occasion of the formal opening of the hospital by King Edward and Queen Alexandra, the latter going round, and in Her usual gracious manner speaking to all the patients, arrived at Miles' cot. As she was admiring his fair curly head, to the amusement of all, the grubby, crumpled picture of King Edward was thrust before Her eyes, and the child said, "Look, Queen! I kept it to show you!" Her Majesty was quite touched, and insisted on telling the King, after an explanation had been given by the embarrassed sister.

